# Real-Time Remote Monitoring of Temperature and Humidity Within a Proton Exchange Membrane Fuel Cell Using Flexible Sensors

**DOI:** 10.3390/s110908674

**Published:** 2011-09-08

**Authors:** Long-Sheng Kuo, Hao-Hsiu Huang, Cheng-Hao Yang, Ping-Hei Chen

**Affiliations:** Department of Mechanical Engineering, National Taiwan University, Taipei, Taiwan; E-Mails: d94522017@ntu.edu.tw (L.-S.K.); r98522115@ntu.edu.tw (H.-H.H.); d95522002@ntu.edu.tw (C.-H.Y.)

**Keywords:** flexible humidity microsensor, flexible temperature microsensor, radio frequency module, power density

## Abstract

This study developed portable, non-invasive flexible humidity and temperature microsensors and an *in situ* wireless sensing system for a proton exchange membrane fuel cell (PEMFC). The system integrated three parts: a flexible capacitive humidity microsensor, a flexible resistive temperature microsensor, and a radio frequency (RF) module for signal transmission. The results show that the capacitive humidity microsensor has a high sensitivity of 0.83 pF%RH^−1^ and the resistive temperature microsensor also exhibits a high sensitivity of 2.94 × 10^−3^ °C^−1^. The established RF module transmits the signals from the two microsensors. The transmission distance can reach 4 m and the response time is less than 0.25 s. The performance measurements demonstrate that the maximum power density of the fuel cell with and without these microsensors are 14.76 mW·cm^−2^ and 15.90 mW·cm^−2^, with only 7.17% power loss.

## 1. Introduction

The development of green energy has been continually emphasized recently due to increasing environmental protection consciousness. The fuel cell is one possible substitute since it produces no carbon dioxide but only water. Fuel cells can be characterized into several categories [1] such as alkaline fuel cells (AFC), phosphoric acid fuel cells (PAFC), solid oxide fuel cells (SOFC), direct methanol fuel cells (DMFC), and proton exchange membrane fuel cells (PEMFC). Among these kinds of fuel cell, PEMFC is widely used because of its compact size, light weight, low operating temperature, and high efficiency [2]. Two kinds of water transport mechanism exist in the proton exchange membrane [3,4]: one is electro-osmotic drag and the other is back diffusion. If these two operating mechanisms are unbalanced, flooding will occur at the anode or cathode flow channels so as to make cell voltage deteriorate. Therefore, it is crucial to build a way of detecting the flooding situation within fuel cells.

Several researchers have developed methods for inner flooding detection. Nishikawa *et al*. [5] drilled holes in bipolar plates, placing a commercial humidity-temperature sensor at the opened location to measure the local humidity. Lee *et al*. [6] manufactured humidity and temperature microsensors on metallic bipolar plates by microelectromechanical systems (MEMS) technology to monitor the moisture state. Although the above methods are distinctive, there are still some problems. For example, Nishikawa’s method may encounter gas leakage and flow channel damage problem, while the measurement position about Lee’s method would be hardly changeable.

Consequently, this study proposes flexible thin-film humidity and temperature microsensors embedded in PEM fuel cell for the purpose of preventing the above issues. In addition, this work also establishes a wireless module to integrate sensors because it has advantages of portability and dependability. Wireless sensors are generally smaller in size and lighter in weight so that it can be carried more easily than conventional instruments. Hence, the investigation employs wireless module to integrate microsensors.

The remaining sections are organized as follows: humidity microsensor, temperature microsensor, and wireless module are introduced first. Then, calibration for sensors, the result of wireless integration and measurement consequence within fuel cell are presented.

## 2. Experimental Setup

### 2.1. Humidity Microsensor

**Figure 1.**
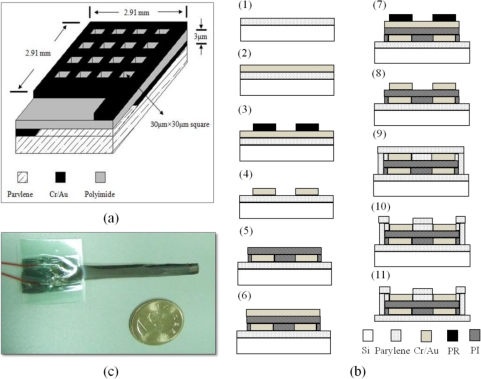
(**a**) A schematic view of designed capacitive humidity microsensor; (**b**) MEMS fabrication process for the humidity microsensor; and (**c**) Photograph of the humidity microsensor.

Humidity microsensors can be categorized into resistive and capacitive types [7,8], according to their output signals. In general, the capacitive humidity sensor is less affected by the temperature variation compared with the resistive humidity sensor. Therefore, in this investigation, a capacitive humidity sensor was used as our sensor. Figure 1(a) shows our designed parallel-type capacitive microsensor. Parylene is employed as the flexible substrate of sensor and polyimide is used as the dielectric substance due to its high sensitivity to moisture [9]. Electrode area is 2.91 mm × 2.91 mm and moisture layer is 3 μm thick, and its top electrode is designed with a number of 30 μm × 30 μm square holes in order to let sensing layer easily adsorb water vapor.

Figure 1(b) displays the MEMS fabrication flowchart of the flexible humidity microsensor. The process begins with the evaporation of a 7.5 μm thick layer of parylene on silicon (Section 1) as the flexible substrate of humidity sensor. Then, a 60 nm thick layer of Cr and a 250 nm thick layer of Au are deposited on parylene substrate using electron beam evaporator (Section 2) and wet-etching method is used so that the structure of bottom electrode is carried out (Sections 3 and 4). To form the pattern of sensing layer, photolithography process is employed as a result that a thickness of 3 μm polyimide is placed on the bottom electrode (Section 5). After finishing the configuration of the sensing layer, Cr and Au are deposited again on the sensing layer and the wet-etching method is used afterwards to define the structure of the top electrode (Sections 6 to 8). At the final step, a 3.75 μm layer of parylene is placed on the top electrode in order to protect the sensor and a reactive ion etching (RIE) system is utilized to open the sensing area to adsorb water vapor (Sections 9 and 10). Finally, the flexible sensor is peeled off from the silicon substrate (Section 11). Figure 1(c) displays the product of finished humidity microsensor.

### 2.2. Temperature Microsensor

**Figure 2.**
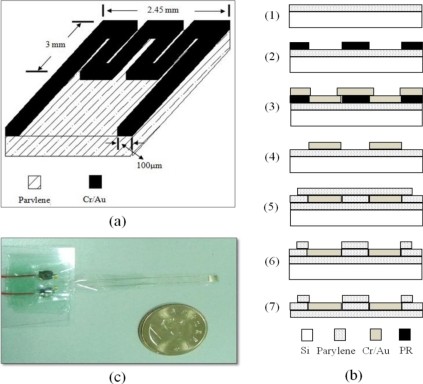
(**a**) A schematic view of designed resistive temperature microsensor; (**b**) MEMS fabrication process for the temperature microsensor; and (**c**) Photograph of the temperature microsensor.

Temperature microsensors are mainly classified into two kinds: thermocouples and resistive temperature sensors. Due to the reason that the fabrication process of thermocouples is complicated so as to increase cost and that resistive temperature sensor shows fine sensitivity, a resistive type sensor acted as our temperature sensor. Figure 2(a) displays the designed temperature microsensor. Its structure is a kind of serpentine resistor whose sensing area is 2.45 mm × 3 mm.

Figure 2(b) exhibits the MEMS procedure for the temperature microsensor, it starts with a 7.5 μm layer of parylene deposited on a silicon substrate (Section 1). Then a photolithography process is employed in attempt to form the pattern of the resistive temperature sensor (Section 2). After finishing the photolithography procedure, a 60 nm thick layer of Cr and a 250 nm thick layer of Au layer are vaporized on the pattern by using an electron beam evaporator apparatus and the remaining photoresist is stripped afterwards so that the structure of temperature sensor is completed (Sections 3 and 4). Moreover, parylene is deposited again so as to protect the temperature sensor. At last, the sensing area is opened using an RIE system to make it detect temperature variation (Sections 5 and 6). The last step is to peel off the flexible sensor from the silicon substrate (Section 7). The product of completed temperature microsensor is shown in Figure 2(c).

### 2.3. Wireless Module

**Figure 3.**
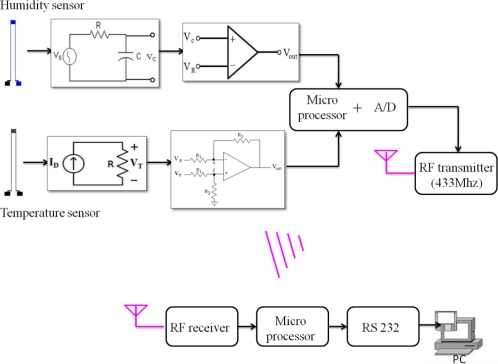
Block diagram of the wireless sensing system.

Figure 3 exhibits the block diagram of the established microsensors integrated with wireless module. It is composed of the sensing circuits for each microsensor, microprocessor, and transmitter. The content of the sensing circuit for capacitive humidity sensor is that an input signal from a square wave oscillator transmits to RC circuit where C is the capacitive sensor, when the capacitance change happens, the time constant of output signal from sensor varies consequently. Then, this signal transmits to OP comparator and a microprocessor counts the time that the signal requires to reach the reference voltage. The sensing circuit for resistive temperature sensor is that a current source signal transfers to the resistive temperature sensor so that its output voltage signal changes with environmental temperature fluctuation. The output signal is dispatched to a differential amplifier circuit to make signal amplification and offset reduction, and the revised signal transmits to a microprocessor afterwards, whose ADC range is 0 to 3 V. When a microprocessor gets signals from each sensor, it transfers them to RF transmitter which operates at 433 MHz. Afterwards, wireless receiver takes in signals, transmitting them to a microprocessor so as to make data processing and to show them in display interface.

## 3. Results and Discussion

### 3.1. Calibration and Wireless Integration

**Figure 4.**
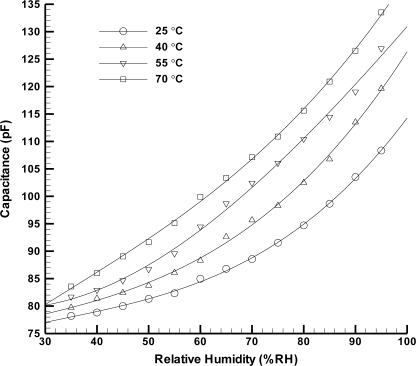
Capacitive humidity microsensor performances at different temperatures in an environmental chamber.

The humidity microsensor is wire bonded using a dispenser and is tested inside an environmental chamber (Model HUNGTA-HT-8045A). Capacitance measurements were performed at 1 kHz using a LCR meter (Model Wayne Kerr 4230). Tests for the humidity sensor are conducted at various temperatures from 25 °C up to 70 °C as well as for different relative humidity levels ranging from 35%RH to 95%RH. The capacitance value of the microsensor is always recorded after waiting for 15 min at each measurement condition to assure that the whole volume of the climatic chamber reaches a stable temperature and humidity level. Figure 4 shows the humidity microsensor performance. One can see that the sensitivity of the humidity microsensor (defined by α_H_ = ΔC/Δ%RH [10]) increases when the environmental temperature rises. This is because the thermal expansion causes the enlargement of electrode area so that the capacitance of sensor increases. The sensitivity of capacitive humidity sensor at best is 0.83 pF%RH^−1^ at 70 °C. In addition, the linearity gets better as the temperature increases. Table 1 exhibits a comparison between the sensitivity of capacitive humidity sensors reported in the literature [11–13], and the sensitivity of sensor in this work demonstrates excellent result.

**Figure 5.**
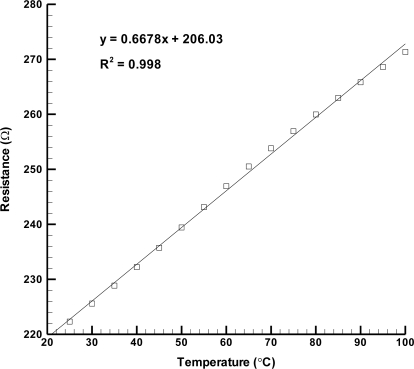
Resistive temperature microsensor performance.

In the calibration of the temperature microsensor, it is also characterized in the same environmental chamber. Resistance measurements are made through a digital multi-meter (Model HP-HEWLETT-PACKARD-3478A). According to the reference [14], the sensitivity of resistive temperature sensor is described as:

(1) $$\alpha =\frac{R_{a}-R_{b}}{R_{b}\Delta T}$$

where R_a_ is resistance value at temperature a °C, R_b_ is the resistance value at temperature b °C, ΔT is the temperature difference (=a − b). Figure 5 displays the sensor performance with the sensitivity of 2.94 × 10^−3^ °C^−1^. In comparison with other works [15–17] as shown in Table 2, this resistive temperature sensor achieves remarkable performance. The linear regression of sensor is 0.998, which demonstrates an excellent value.

**Figure 6.**
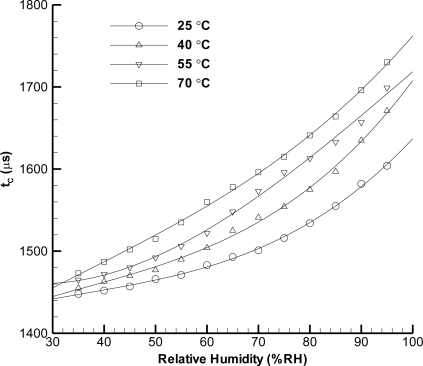
Calibration of wireless integration for humidity sensor at different temperatures (25, 40, 55, 70 °C) in environmental chamber.

**Figure 7.**
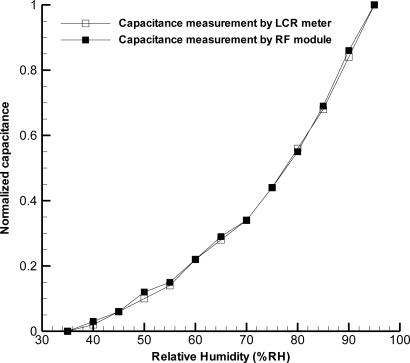
Comparison between capacitance measurement by LCR meter and by RF module at the temperature of 25 °C.

**Figure 8.**
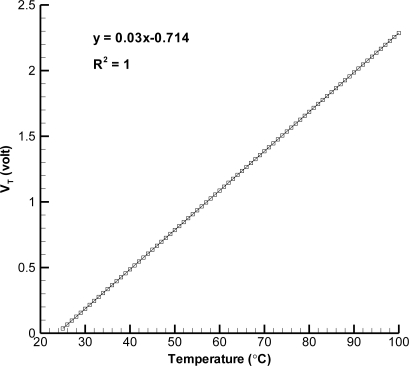
Calibration of wireless integration for temperature sensor.

The measurement results by wireless integration are displayed in Figures 6 to 8. Figure 6 shows the time constant measurement (=RC), which is processed by the microprocessor in the RF module. In order to fairly compare the results between capacitance measurement by LCR meter and RF module, we define a normalized factor C_n_ as follows:

(2) $$C_{n}\left(T\right)=\frac{C\left(T\right)-C_{\text{min}}\;\left(T\right)}{C_{\text{max}\;(T)-C_{\text{min}}\;\left(T\right)}}$$

Figure 7 shows that two sets of measurement data are very consistent, indicating that the processing and 8-bit resolution of RF module did a good job.

Figure 8 exhibits the voltage measurement of wireless integration of temperature sensor (through microprocessing and A/D conversion) and it displays that the linear regression of sensor is almost 1, which is close to that of temperature sensor performance. The resolution of temperature sensor is 0.1 °C. Moreover, the wireless measuring distance is 4 m at most and its response time is less than 0.25 s by the test.

### 3.2. Diagnosis within Fuel Cell

**Figure 9.**
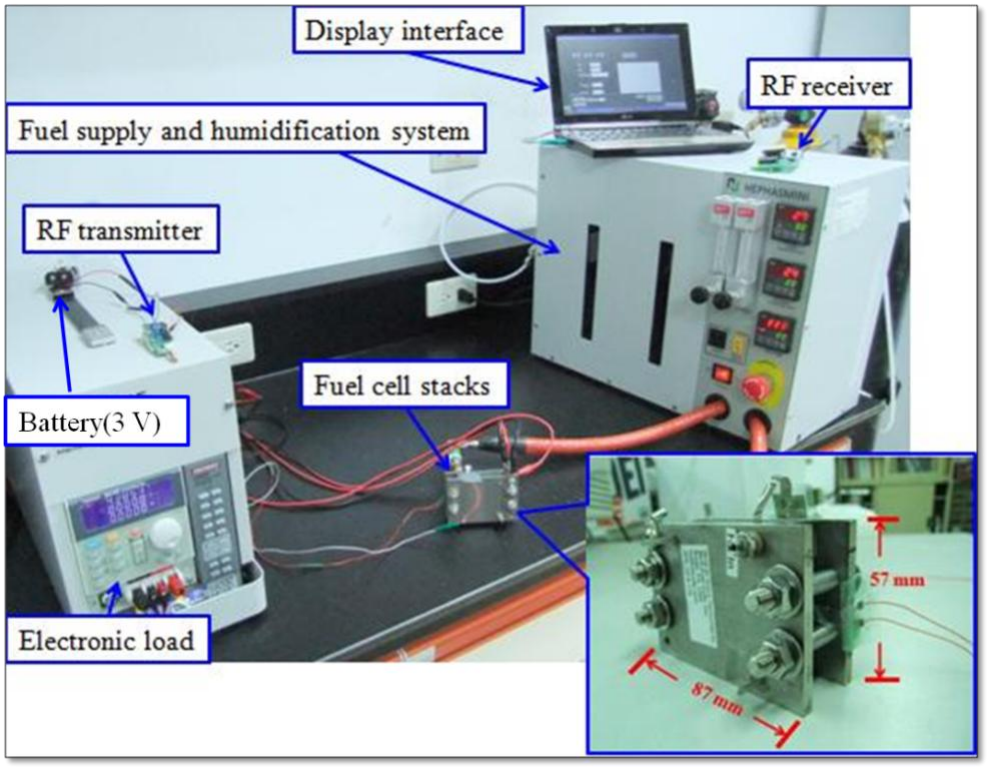
Photo of fuel cell operating system and wireless sensing system.

**Figure 10.**
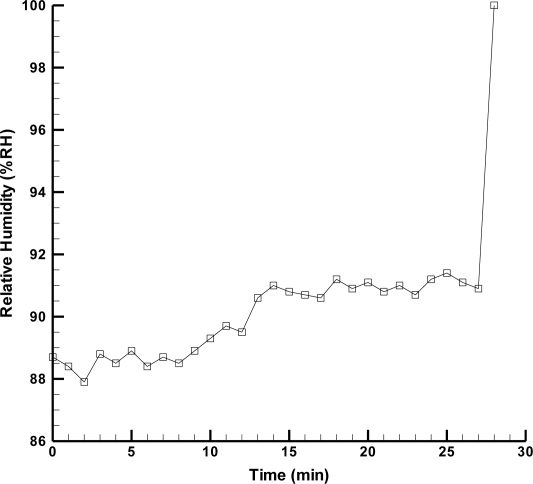
The humidity diagnosis within fuel cell using wireless sensing system

**Figure 11.**
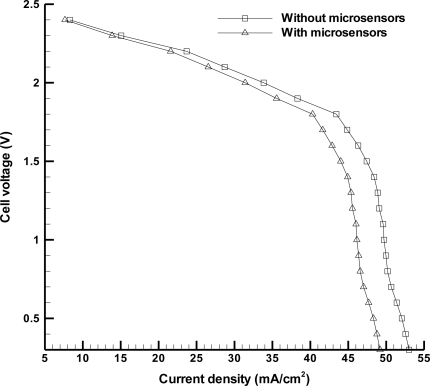
Fuel cell performance with and without microsensors.

The wireless sensing system is implemented to perform internal diagnosis of a fuel cell. Figure 9 displays the experimental setup of the wireless sensing system and the fuel cell operating system. The fuel supply and humidification equipment provides humidified fuel gas for the three fuel cell stacks. When it begins to generate power as a result of getting fuel gas, the electronic load records the voltage condition of fuel cell and the wireless sensing system detects the internal status of the fuel cell during operation. Figure 10 exhibits the local humidity detection at the anode flow channel inlet within a fuel cell whose operating conditions are a fuel flow rate of 60 mL min^−1^ and humidification temperature of 70 °C. Flooding is observed after steady output voltage for 28 min. Thus, it is necessary to revise the humidification condition for the fuel cell to improve this situation. Figure 11 demonstrates the performance of fuel cell with and without microsensors. The maximum power density of the fuel cell with and without microsensors are respectively 14.76 mW·cm^−2^ and 15.90 mW·cm^−2^, indicating the power loss ratio of 7.17%. Such the power loss ratio is much better than 55% of Lee [18].

## 4. Conclusions

This study has successfully established a distinctive means for measuring the internal status of a PEMFC. A thin film and flexible capacitive humidity microsensor and a resistive temperature microsensor are fabricated using MEMS processing. The humidity sensor can reach a sensitivity of 0.83 pF%RH^−1^ and the temperature sensor exhibits a sensitivity of 2.94 × 10^−3^ °C^−1^. Furthermore, a portable, lightweight wireless RF module is developed to transmit the sensor signals. Such the design enables the diagnosis of a fuel cell out of the lab. The wireless detection distance reaches 4 m and its response time is less than 0.25 s. The performance tests show that the maximum power density of the fuel with and without microsensors are 14.76 mW·cm^−2^ and 15.90 mW·cm^−2^. In other words, the power density loss ratio is only 7.17%. With regard to further applications of the wireless sensing system, it can be integrated with humidification control equipment to achieve the formation of closed loop system so as to carry out the aim of *in situ* gas humidification management.

## Figures and Tables

**Table 1.** Comparison of sensitivity for different capacitive humidity sensors using polyimide presented in the literature.

**Reference**	**Best sensitivity**
Gu *et al*. [11]	5 fF%RH^−1^
Kim *et al*. [12]	0.78 pF%RH^−1^
Kim *et al*. [13]	0.35 pF%RH^−1^
This study	0.83 pF%RH^−1^

**Table 2.** Comparison of sensitivity for different resistive temperature sensors presented in the literature.

**Reference**	**Electrode material**	**Sensitivity**
Xiao *et al*. [15]	Pt	2.91 × 10^−3^ °C^−1^
Shih *et al*. [16]	Pt	2 × 10^−3^ °C^−1^
Innovative Sensor Technology [17]	Pt	3.85 × 10^−3^ °C^−1^
This study	Au	2.94 × 10^−3^ °C^−1^
